# Orthodontic bonding and debonding induces structural changes but does not alter the mechanical properties of enamel

**DOI:** 10.1186/s40510-018-0211-7

**Published:** 2018-05-07

**Authors:** Alexis Ioannidis, Spyridon N. Papageorgiou, Iosif Sifakakis, Spiros Zinelis, George Eliades, Theodore Eliades

**Affiliations:** 1 Clinic of Fixed and Removable Prosthodontics and Dental Material Science, Center of Dental Medicine, University of Zurich, 8032 Zurich, Switzerland; 2 Clinic of Orthodontics and Pediatric Dentistry, Center of Dental Medicine, University of Zurich, 8032 Zurich, Switzerland; 3 Department of Orthodontics, School of Dentistry, National and Kapodistrian University of Athens, 115 21 Athens, Greece; 4 Department of Biomaterials, School of Dentistry, National and Kapodistrian University of Athens, 115 21 Athens, Greece

**Keywords:** Dental enamel, Acid etching, Dental bonding, Composite resin, Orthodontics

## Abstract

**Background:**

The purpose of the study is to investigate the effect of the bonding procedure on the mechanical properties of human enamel.

**Methods:**

A total of 20 extracted human premolars were included in this study, with the half of each tooth acting as its own internal control. Embedded and horizontally cut specimens were prepared, and two bucco-orally zones were separated. The first enamel zone of each tooth remained untreated. The opposing zone was subjected to simulated bonding and debonding, including etching with 37% phosphoric acid, bonding with primer and flowable composite resin, and subsequent removal of the composite with an adhesive removal bur. The properties tested were (a) elemental composition by energy-dispersive X-ray analysis, (b) mechanical properties of specimens by instrumented indentation testing (Martens hardness, elastic modulus, and elastic index), (c) enamel morphology by low-vacuum scanning electron microscopy, and (d) molecular composition by Raman microspectroscopy. Statistical analysis was performed by one-way mixed-effects analysis of variance at *a* = 0.05.

**Results:**

No significant differences could be found in the mechanical properties (Martens hardness, elastic modulus, and elastic index) and elemental composition of intact and treated enamel, with the possible exception of Si traces, which were found only in the latter. Raman analysis revealed no differences between the two surface states while shallow grooves and parallel lines were found on the surface of treated enamel by SEM analysis.

**Conclusions:**

Under the limitations of the conditions of the study, there were no mechanical properties’ alterations on enamel subjected to orthodontic bonding.

## Background

Although bonding to tooth tissues has long been of particular interest in dentistry, the introduction of the acid etch method by Buonocore was revolutionary [1]. This technique enabled through the inherent enamel’s anatomy the utilization of adhesive penetration in terms of resin tags [2] in order to increase retention of the bonded material to the tooth. Therefore, the acid etch technique has found numerous applications in almost all aspects of contemporary dentistry, including operative dentistry [3], prosthodontics [4], pediatric dentistry [5], and orthodontics [6]. Although this technique has been widely adopted in all these dental fields, it is not free of complications and adverse consequences. Acid conditioning of dental enamel causes superficial tissue loss that ranges between 20 and 50 μm [7], while a possible removal of the bonded material or the bonded attachment (de-bonding) is associated with additional loss of dental tissue [7], enamel tear-outs [8], or enamel cracks [8].

The adverse effects of the acid etch bonding protocol are of particular importance in orthodontics for several reasons. First, a substantial percentage of orthodontic patients are in their adolescent life period, do not have an acute pathology, and seek treatment for esthetic, functional, or psychosocial reasons [9]. Therefore, the idea of permanent adverse effects on their dental tissues might be viewed with trepidation. Second, it is not uncommon for orthodontic patients to have several brackets being rebounded during treatment owing to bracket-enamel failure during mastication, a fact which implies that these patients experience additional loss of tooth substance. Additionally, the macro- and micro-tags of the resin adhesive penetrate the conditioned enamel layer to depths reaching 50 μm [10], and thus, the removal of the resin after the completion of treatment is not possible [12] and this adhesive remnant might lead to enamel discoloration [11]. Also, post-debonding protocols involving removal of adhesive residuals with various rotary abrasive tools or hand instruments may increase the roughness of the enamel surface [12] and may further lead to color alterations [13].

The foregoing discussion emphasizes the importance of the assessment of structural, compositional, and mechanical effects of etching and bonding on uncut enamel. Therefore, aim of the present study was to assess the mechanical and chemical alterations of dental enamel that has been treated with a simulated bonding protocol of acid etching and bonding and to compare its sound enamel. The null hypothesis tested was that there is no difference in all aforementioned properties between untreated and treated enamel.

## Methods

### Specimen preparation

In total, 20 human premolars, all extracted due to orthodontic reasons, were cleaned and stored in deionized water. Teeth were anonymized by securing that no tracking of the donor could be made through storage (all teeth were stored as they were obtained in a single source). The Ethics Committee of the School of Dentistry of National and Kapodistrian University of Athens has determined that under these conditions, no ethical approval is necessary for performing studies on extracted human teeth.

**Fig. 1:**
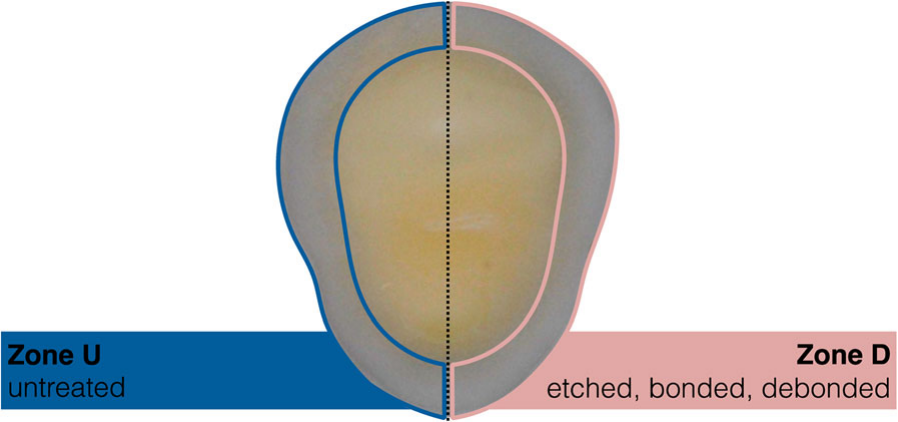
Schematic drawing of the specimens prepared. The Zone U (untreated) and the Zone D (etched, bonded, debonded) are easily distinguished by the vertical line

All selected teeth were free of fillings, surface fractures, or decays. The roots of the teeth were horizontally cut approximately 2~4 mm beneath the cemento-enamel junction with a diamond bur. Thereafter, the specimens were embedded in cylindrical molds (∅ 25 mm) in a self-curing epoxy resin (Caldofix, Struers, Ballerup, Denmark) with the apical flat-cut surface facing downwards. After curing for 1.5 h under heat (75 °C), the specimens were cut along their mesial-distal orientation. Then, the specimens were metallographic ground and polished in a grinding polishing machine (Dap-V, Struers). The specimens were ground employing SiC water coolant papers (220 to 4000 grit) and polished using a 0.04-μm colloidal silica suspension (OP-U, Struers). Then, the exposed horizontally tooth surfaces were bucco-orally separated into two zones with a sharp line cut (Fig. 1).

### Simulated bonding and debonding procedures

In all specimens, one enamel zone remained untreated. The enamel zone of treated specimens was etched for 30 s with 37% phosphoric acid (Pegasus; Astek Innovations Ltd., Altrincham, UK) and consecutively sprayed with water for another 30 s. After drying the specimens with airflow, a bonding agent (OrthoSolo Universal Bonding Enhancer; Ormco Corp., Glendora, CA, USA) was applied for 30 s and blown carefully with air. At the same location, a flowable composite material (Enlight Light Cure Adhesive; Ormco Corp., Glendora, CA, USA) was applied and light-cured (radii pus LED curing light, 970 mW/cm^2^; SDI limited, Bayswater, Australia) for 30 s. Thereafter, the composite was cautiously removed with an adhesive removal 18 fluted bur (Renew finishing system; Reliance Orthodontic Products Inc., IL, USA) until the enamel was again exposed. Enamel was finally ground and polished by renew finishing system points and orthodontic prophy pumice.

### Mechanical properties (instrumented indentation testing)

**Fig. 2:**
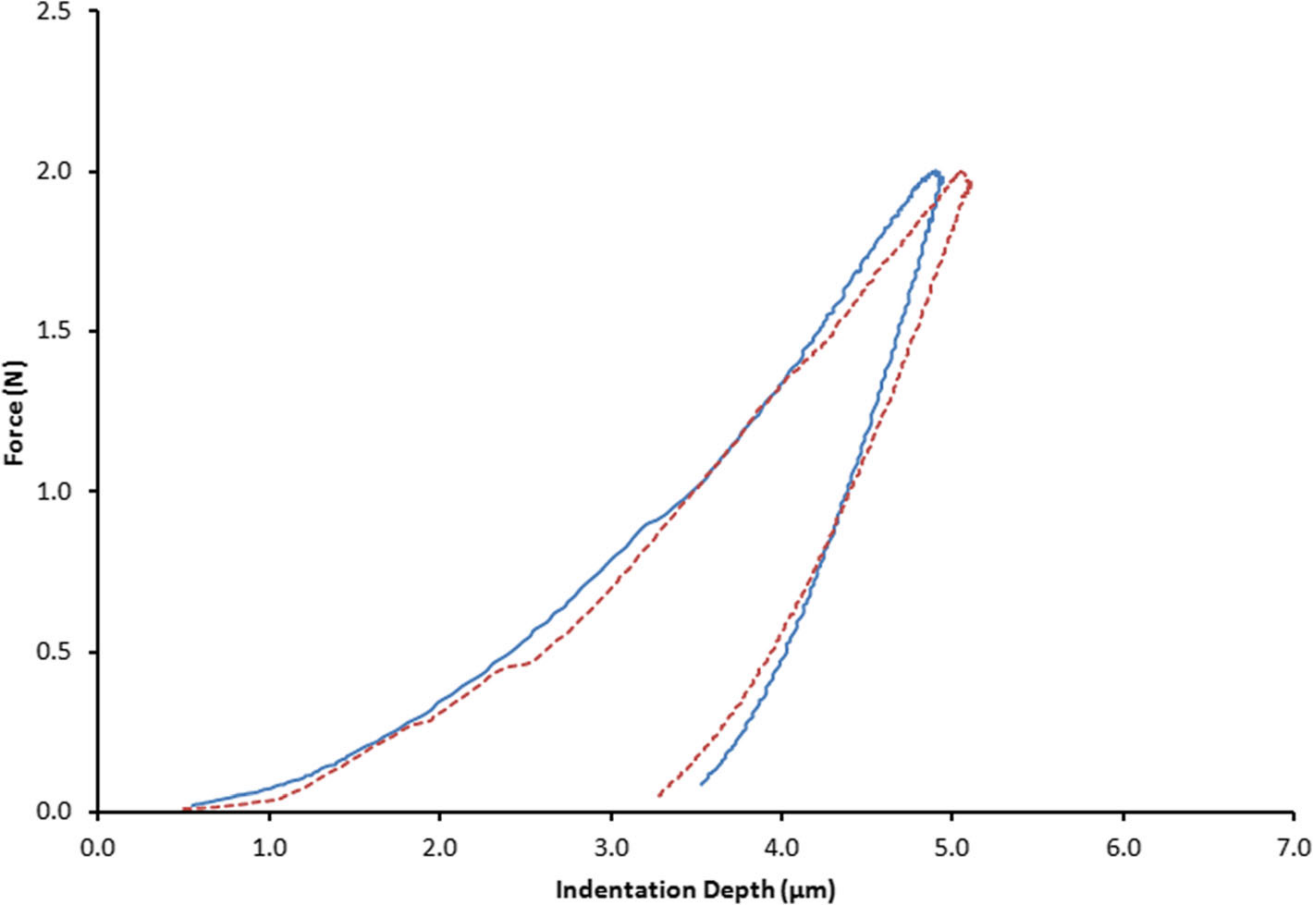
Representative force-indentation depth curves. The shallower indentation depth (continuous line) indicates higher hardness while the steeper unloading curve shows increased indentation modulus of elasticity (continuous line)

Instrumented indentation testing (IIT) measurements were carried out with a universal hardness-testing machine (ZHU0.2/Z2.5; Zwick Roell, Ulm, Germany). Force-indentation depth curves were monitored applying 0.2 N with a 2-s dwell time by a Vickers indenter. On each of the 20 specimens, four curves at each zone were taken almost 100 μm from the outer border of the embedded teeth. The mean value was used as representative for intact and debonded zone. Force-indentation depth curves were recorded (Fig. 2), and the Martens hardness (HM), the indentation modulus (E_IT_), and the percentage of the elastic part of indentation work (%), known as elastic index (η_IT_), were measured according to the ISO 14577-1 [14]. The Poisson’s ratio values were set at 0.33 for enamel.

### Low-vacuum scanning electron microscopy EDX

Of each group, five specimens were selected to analyze the morphology of the untreated and treated enamel zones by means of low-vacuum scanning electron microscopy (LV-SEM) imaging. Secondary electron images and backscattered electron images were acquired with a SEM unit (Quanta 200; FEI, Hillsboro, OR, USA), working under the following conditions: 25.0 kV accelerating voltage 90 μA beam current, approximately 1.0 Torr pressure and × 600 nominal magnification.

The elemental composition of the different zones was investigated using energy-dispersive X-ray (EDX) spectroscopic analysis. Three spectra per specimen were collected using an XFlash 6|10 silicon drift detector (Bruker, Berlin, Germany) with a slew window under the same operating conditions: a 210 x 210 μm sampling window and a 200-s acquisition time. The quantification was carried out in a standard less mode using atomic number, absorbance, and fluorescence correction factors with the dedicated software (ESPRIT version 1.9; Bruker) for the elements Cl, Ca, O, Na, P and Si.

### Raman microspectroscopy

Five randomly selected teeth were further analyzed by Raman microspectroscopy to investigate possible chemical alterations on treated surfaces. One spectrum from each region was acquired with a Raman spectrometer (EZRaman-I, Enwave Optronics, Orange, CA, USA) attached to a microscope (Leica BME, Leica microsystems, Heerbrugg, Switzerland) under the following conditions: 785-nm excitation laser, 380-mW power, 1200–300 cm^−1^ wavelength range, 6 cm^− 1^ resolution. Spectra were acquired and baseline corrected by EZRaman Reader (ver 8.2.8) software (Enwave Optronics, USA).

### Statistical analysis

After data of HM, E_IT_, and η_IT_ were checked for normality and found to be not normally distributed, descriptive statistics were calculated including medians and interquartile ranges (IQR). Mixed-effects analysis of variance was performed to compare between intact and treated (etched, bonded, and debonded) surfaces, with experimental group and tooth unit as the fixed- and random-effects terms respectively, calculating average differences and their 95% confidence intervals (CIs). A two-side *P* value of ≤ 0.05 was considered significant for all analyses, which were run in Stata SE 10.0 (StataCorp, College Station, TX).

## Results

### Mechanical properties

The median HM for intact and treated enamel specimens were 2866 N/mm^2^ (IQR = 2809 to 3154) and 2989 N/mm^2^ (IQR = 2625 to 3211), respectively, with no significant differences (*P* > 0.05; Table 1). The E_IT_ for intact and treated enamel specimens were 70.3 GPa (IQR = 59.7 to 72.5) and 69.7 GPa (IQR = 66.3 to 77.1), respectively, again with no significant differences (*P* > 0.05). Finally, the same was seen for intact and treated enamel specimens in terms of the η_IT_ with medians 38.3% (IQR = 35.3 to 41.7) and 36.6% (IQR = 33.0 to 39.1), where no significant difference was seen (*P* > 0.05).

**Table 1:** Results for the mechanical properties of the analyzed specimen

		Martens hardness (N/mm^2^)		Indentation modulus (GPa)		Elastic index (%)	
Group	*N*	Median (IQR)	*P* value*	Median (IQR)	*P* value*	Median (IQR)	*P* value*
Intact enamel	20	2866 (2809, 3154)	0.760	70.3 (59.7, 72.5)	0.856	38.3 (35.3, 41.7)	0.272
Treated enamel	20	2989 (2625, 3211)		69.7 (66.3, 77.1)		36.6 (33.0, 39.1)	

*IQR* interquartile range

*From mixed-effects model

### Qualitative LV-SEM image analysis and energy-dispersive X-ray spectroscopic analysis

**Fig. 3:**
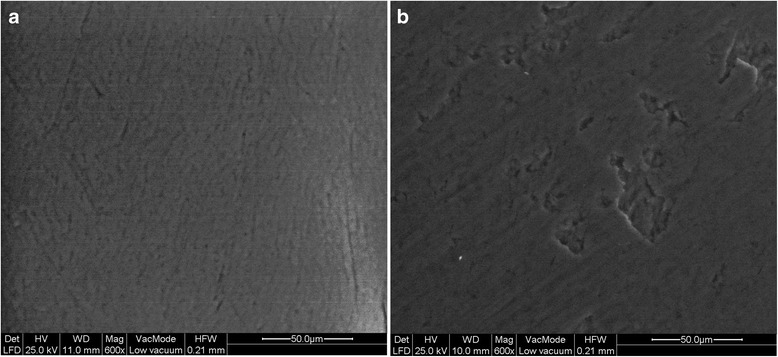
Representative secondary electron images from the surfaces of intact (**a**) and treated (**b**) enamel. A random distribution of shallow grooves is shown along with a few parallel lines (Original magnification × 600)

Low-vacuum secondary electron images of intact and treated enamel are given in Fig. 3. Intact enamel (Fig. 3a) showed a smear-layer-coated surface with distinct polishing patterns and few irregularities like pits, scratches, and prismatic boundaries, while treated enamel demonstrated again a smear-layer coating with scratches, pits, and some prismatic boundaries. Parallel lines were also identified along with a diffuse distribution of shallow grooves (Fig. 3b).

**Fig. 4:**
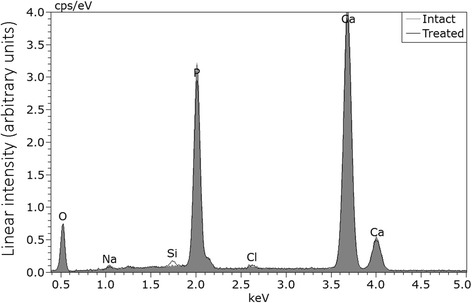
Representative EDX spectra from the surfaces of intact and treated enamel. Gray area stands for intact while black line for treated enamel. Traces of Si were identified only on treated surfaces

Representative energy-dispersive X-ray spectra from the enamel groups tested are presented in Fig. 4, while the quantitative results of energy-dispersive X-ray analysis are given in Table 2. Both intact and treated enamel share similar qualitative and quantitative analysis. The sole exception was Si, where traces were identified only to treated enamel.

**Table 2:** Elemental composition (wt%, mean values) of the enamel surfaces for the intact and treated specimens after energy-dispersive X-ray analysis (*n* = 5)

Element	Intact enamel	Treated enamel
O	43.1	44.1
Na	0.9	0.6
Si	– (BDL)	0.4
P	16.9	16.4
Cl	0.2	0.2
Ca	38.9	38.3

*BDL* below detection limit

### Raman

**Fig. 5:**
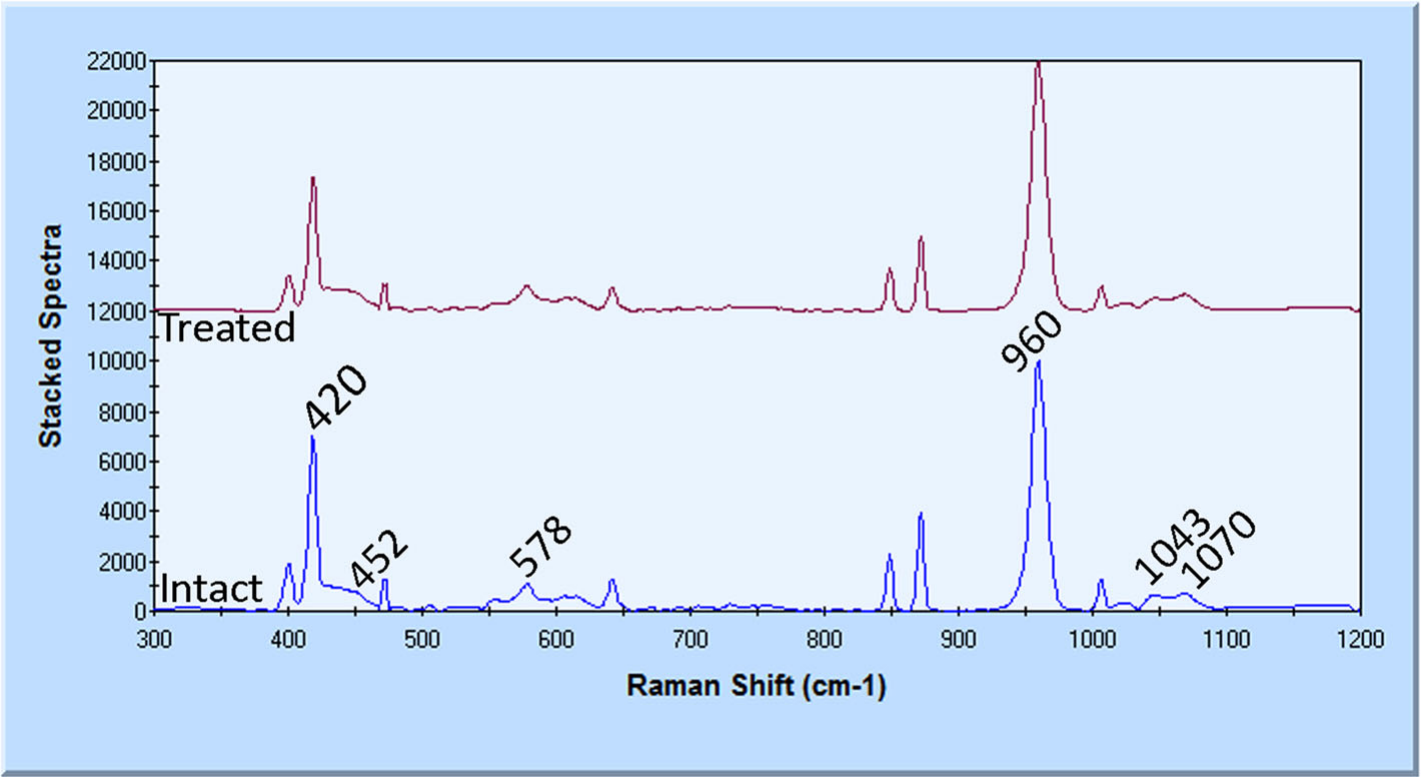
Raman spectra from the surface of intact and treated enamel showing phosphate (420, 452, 578, and 960 cm^−1^) and carbonate (1043 and 1070 cm^−1^) main vibrational modes

Two representative spectra from intact and treated enamel are presented in Fig. 5. Both spectra showed the same intensity providing phosphate and carbonate main vibrational modes, and thus, the tested surfaces should be considered similar. The assignment of inorganic peaks is as follows: 420 and 450 cm^−1^ (v_2_PO_4_^3−^), 578 cm^−1^ (v_4_PO_4_^3−^), 960 cm^−1^ (v_1_PO_4_^3−^), and 1043 and 1070 cm^−1^ (bending and stretching modes of carbonate CO_3_^2−^).

## Discussion

The results of this study indicate that acid etching does not alter significantly the mechanical properties of dental enamel in terms of HM, E_IT_, or η_IT_ and molecular structure according to Raman analysis. Surface characterization demonstrated slight differences while only Si traces were identified on the surface of treated enamel after EDX analysis. In general, both surfaces can be considered as similar in overall and thus the null hypothesis should be predominately accepted.

The analysis with LV-SEM is considered beneficial for this study in order to avoid any possible morphological artifact or elemental interference related to conductive coating procedure. The LV-SEM image analysis indicated similar surfaces characterization between intact and treated enamel with only small shallow grooves seen in the surface of the latter (Fig. 3b). This is in agreement with the literature suggesting the removal of resin remnants during debonding leaves an enamel surface which is similar to the surface of intact enamel [13, 15], although to a small cost loss of dental tissue [13]. Small irregularities in the surface of debonded enamel as were seen in the present study might be appended to bracket debonding procedure (where enamel fragments are removed along with adhesive resin) or to the use of rotating instruments even after careful removal of the adhesive [16]. It is worthwhile to be noted that only minor differences can be seen across the various adhesive removal systems in terms of time needed, adhesive remnants, and amount of enamel loss [7].

Raman analysis did not reveal any qualitative difference in elemental structure between the two surfaces while Si traces found in the treated enamel specimen after EDX analysis should be attributed to either adhesive remnant located possible in the tags or the polishing bur.

Although HM and HV values can be identified simultaneously for the same indentation, only HM values are presented in this study. HM is calculated based by the geometry of Vickers indenter and indentation depth under testing load while Vickers based on the measurement of diagonal length of impression. However as HM is calculated by geometrical data during maximum loading, any interference of visco-elastic properties of material tested and elastic recovery of material around the indentation is minimized. The latter tends to overestimate Vickers hardness. No significant differences in HM were identified, and this finding is in accordance with the results of a previous study comparing intact enamel to enamel treated with a similar acid etch protocol, infiltrated by low-viscosity light-cured resin, where no significant differences in microhardness were found [17]. This can be explained by the fact that no considerable change in the elemental composition of enamel was induced by etching [18], as the mechanical properties of dental enamel are closely associated with its mineral content [19], for example, in cases of white spot lesions, where enamel loses part of its mineral content, leading to a slight reduction in microhardness [17]. This might also explain the increased enamel hardness values that have been reported compared to conventional acid etch protocols with self-etching priming adhesives due to partial reincorporation of mineral into the bond [17, 19]. The mechanical properties tested have different clinical relevance. Hardness in any expression is related with the wear resistance. The higher the hardness, the higher the attrition resistance, and thus, surface integrity can be better preserved during clinical function. Higher elastic modulus indicates that the enamel can withstand higher forces per surface unit preventing catastrophic fracture especially at cervical region where thinner enamel layers are located. Elastic index is indicative of the brittleness of materials tested. In general, brittle materials tend to fracture with ease consuming less fracture energy.

Nanoindentation testing for in depth profiling [20] of bonded enamel has revealed a significant decrease in hardness and elastic modulus only at the outermost layers of 5 μm. Although tiny indentations are beneficial for successive indentations with limited inter-indentations distance such in the case of in depth profile analysis, the results obtained for inhomogeneous materials, such as resin-impregnated enamel surface, may significantly vary across the testing area, and thus, the generalizability of the results may be inaccurate. Another factor, which contributes to the variability of results across studies, may be the great individual quantitative and qualitative variation in the enamel between different patients [21], which can potentially confound research findings. As however, the two halves of the same tooth acted as both experimental and internal control in each group and this was reflected also in the statistical analysis, this is of minor importance.

On the other hand, an experimental limitation of our study was that the quantitative results may be affected by the properties of sound enamel as the thickness of tested materials should be at least 10 times the indentation [22]; this value was estimated to reach 45 μm in this study, which is significantly deeper compared to the nominal thickness of 20 μm of etched enamel. However, despite their different experimental limitations, the aforementioned research protocols concluded that the mechanical properties of treated enamel remain unaffected by orthodontic bonding procedure.

## Conclusions

Under the limitations of the conditions of the study, there were no mechanical properties’ alterations on enamel subjected to orthodontic bonding.
